# Cat-Scratch Disease: Unusual Perivascular Chorioretinal Lesions

**Published:** 2014

**Authors:** Ozlem Sahin

**Affiliations:** Department of Ophthalmology/Uveitis and Ocular Immunology, DunyaGoz Hospital, Ankara, Turkey

**Keywords:** Bartonellahenselae, Cat-scratch Disease, Retinal Vascular Lesions, Chorioretinal Lesions

## Abstract

This study is a case report of bilateral perivascular chorioretinal lesions associated with Bartonella henselae. A 37-year-old woman presented with headache and blurred vision in both eyes aggravating occasionally during five years. She was otherwise healthy, with best-corrected visual acuities were 20/20 in both eyes. History of close contact with cats was more than merely eye-catching upon examination of her fundus. In both eyes, fundi were coated with yellow-brown pigmented perivenous chorioretinal lesions along the superotemporal and inferotemporal vascular arcades and their branches. The perivenous lesions were associated with vascular fibrous bands and corresponding changes in vascular calibers. There were no associated intraocular inflammatory signs in both eyes. The serologic tests confirmed the diagnosis of cat-scratch disease. The patient received no treatment, and she was followed for three years without any signs of ocular inflammation

## INTRODUCTION

*Bartonella henselae* (*B. henselae*) is a fastidious, intracellular, gram-negative alphaproteobacterium that predominantly infects mammalian erythrocytes and endothelial cells, causing long-lasting intraerythrocytic bacteremia (1, 2). *B. henselae* may affect immunocompetent or immunocompromised individuals of all ages. It is known as the causative agent of the cat-scratch disease (CSD) (3). 

The major host reservoirs for *B. henselae* are cats (3). Transmission to humans might occur with contact with cats or cat-related trauma (4). Other possible vectors for *B. henselae* infection are ticks and biting flies (4). Cat-scratch disease is a self-limiting illness characterized by regional lymphadenopathy, fever, and small skin lesions (3). From 5 to 25% of *B. henselae* infections may manifest as a systemic form, with the ocular bartonellosis being the most common systemic manifestation (5). 

The most common ocular presentation is reported as the part of Parinaud’s oculoglandular syndrome, the unilateral neuroretinitis (5). Other ocular presentations include neuroretinitis, retinochoroiditis, retinal vascular occlusions, vasculitis, vitritis, anterior uveitis, intermediate uveitis, and posterior uveitis (6-10). The purpose of this study is to describe the unusual perivascular chorioretinal lesions associated fibrous bands along the intraocular vessel walls; highlighting the changes in vascular caliberswithout any signs of intraocular inflammation.

## CASE REPORT

An otherwise healthy 37-year-old woman presented with headache and occasionally aggravated blurred vision in both eyes lasting for five years. 

**Figure 1:**
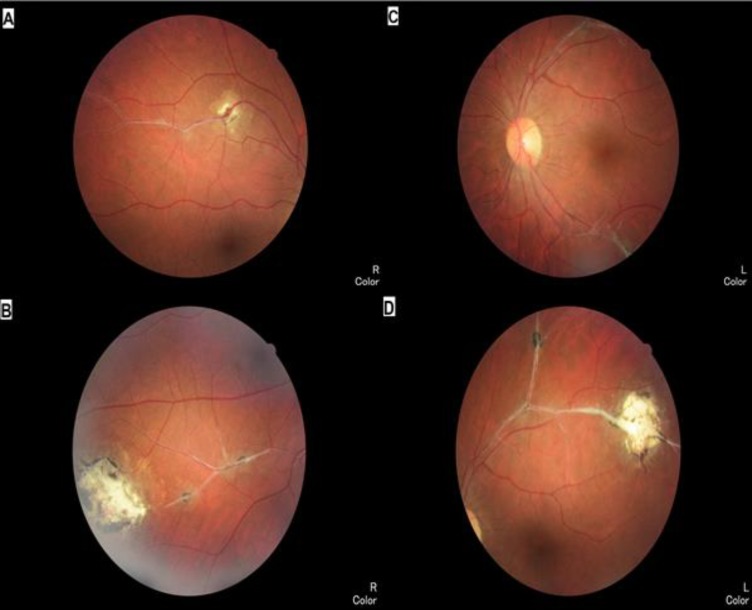
Color fundus photographs of the right (A), (B) and the left (C), (D) eyes displaying yellow-brown perivascular lesions ranging from less than ½ to 2 DD in size down the superotemporal and inferotemporal vascular arcades, coupled by fibrous bands along the vessel walls.

Her best-corrected visual acuities (BCVA) were 20/20 in both eyes and bilateral intraocular pressures (IOP) were normal. Anterior segments were uneventful in both eyes with no intravitreal cells. Fundus examination of the right eye revealed a yellow-brown perivascular lesion lesser than1/2 of disc diameter (DD) in size along the superotemporal arcade. This lesion was associated with fibrous bands along the vessel wall (Fig. 1A). Another lesion was also yellow-brown perivasculary located, ranging from less than ½ up to 2 DD in size, along the inferotemporal vascular arcade associated with fibrous bands along the vessel wall (Fig. 1B). Fundus examination of the left eye disclosed fibrous bands along the superotemporal and inferotemporal vascular arcades (Fig. 1C), and yellow-brown pigmented perivascular chorioretinal lesions less than ½ and 2 DD in size along the superotemporal vascular arcade associated with fibrous bands along the vessel wall (Fig. 1D). In the anamnesis, she mentioned history of close contact with cats, although, no cat-related trauma. 

**Figure 2:**
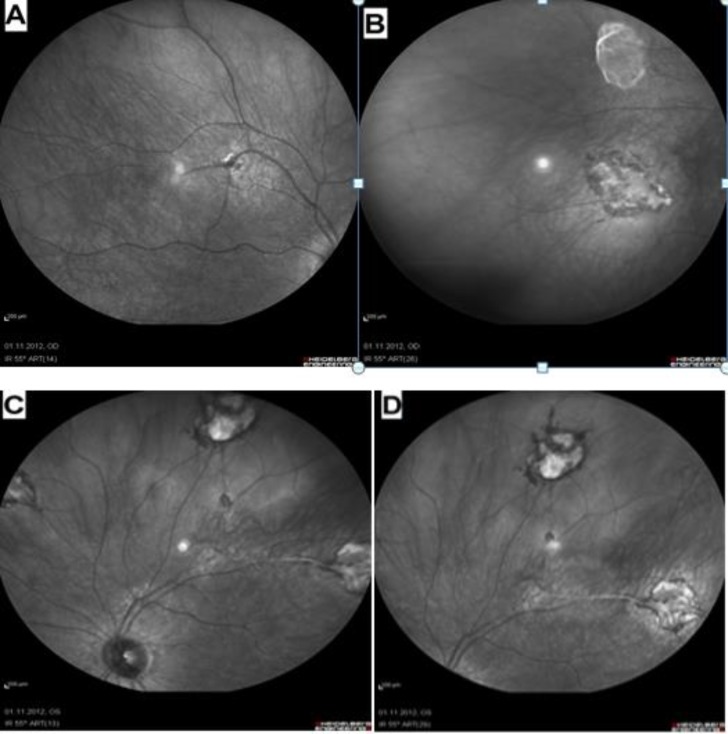
Fundus autofluorescence of the right (A), (B) and the left (C), (D) eyes showing the homogenous increase in autofluorescence corresponding to the perivascular chorioretinal lesion areas.

**Figure 3:**
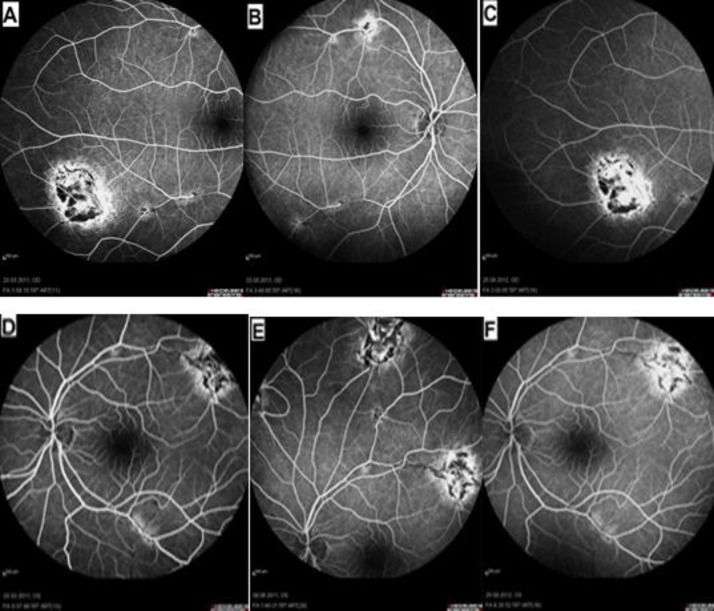
Fundus fluorescein angiography of the right (A), (B) and (C), as well as the left (D), (E) and (F) eyes disclose perivenous location of the lesions along the superotemporal and inferotemporal vascular arcades associated with staining both at arteriovenous, (A, B, D, E) and late venous phases (C, F) in both eyes. Changes in vascular calibers along the inferotemporal vascular arcade,(A) and superotemporal vascular arcade (B) in the right eye, changes in vascular calibers along the inferotemporal arcade, and vascular occlusion along the superotemporal arcade (D, F) associated with loop vessel formation (E) in the left eye are disclosed.

Fundus autofluorescence (FAF) of the right eye revealed a homogenous increase in autofluorescence corresponding to the chorioretinal lesions along the superotemporal and inferotemporal vascular arcades (Fig. 2 A, B). Fundus autofluorescence of the left eye revealed a homogenous increase in autofluorescence corresponding to the chorioretinal lesions along the superior vascular arcades (Fig. 2 C, D).Fundus fluorescein angiography (FFA) of the right eye showed perivenous location of the lesions commencing with appearance at arteriovenous and late venous phases indicating changes in vascular calibers around the lesions (Fig. 3 A, B, C). Fundus fluorescein angiography of the left eye revealed perivenous lesions beginning to appear at arteriovenous and late venous phases (Fig. 3 D, E, F), establishing changes in vascular caliber (Fig. 3 D ,F) with formation of vascular loops along the superonasal vascular branch (Fig. 3 E).

**Figure 4:**
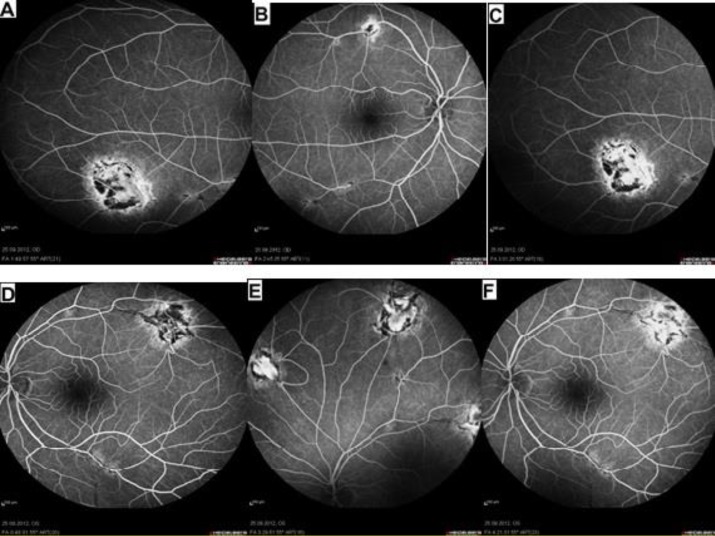
Fundus fluoresce in angiography of the right (A), (B) and (C), and theleft (D), (E) and (F) eyes at the 3^rd^ year of follow-up. Note the stability of inactive perivenous chorioretinal lesions in both eyes.

Down the superotemporal vascular arcade occlusion of the blood flow could be seen around the lesion (Fig. 3 E, F). There was no leakage at the macula at arteriovenous and late venous phases in both eyes (Fig. 3 A, B, D, E, F).

The investigative serological tests were negative except for a positive serological titer for *B. henselae* Ig G which was positive over 1:256 dilution, and a negative titer for *B. henselae* Ig M. Thus, the patient was diagnosed as having bilateral perivenous chorioretinal lesions with vascular fibrosis and occlusion associated with CSD. The patient was followed for three years without treatment, and neither activation of the lesions on FFA (Fig. 4 A-F), nor were intraocular inflammatory signs observed during her follow up in both eyes.

## DISCUSSION

Posterior segment manifestations of ocular bartonellosis include neuroretinitis, intermediate uveitis, focal retinal vasculitis, retinitis, branch retinal arteriolar or venular occlusions, vitreous hemorrhage, focal choroiditis, serous retinal detachments and peripapillary angiomatous lesions (11-15). Discrete white retinal or choroidal lesions ranging from 1/6 to 2 DD, and well-defined retinal opacifications with features of multiple retinal arteriolar occlusions have been considered as rare posterior segment manifestations of ocular bartonellosis (9). 

Vascular-occlusive events have been revealed at the site of the chorioretinal lesions (9). Our case had bilateral prominent perivenous chorioretinal lesions ranging ½ to 2 DD associated with perivenous fibrosis and occlusions of the vessels. Severe retinal phlebitis with venular occlusions has been reported in ocular bartonellosis (16). However, our case had no signs of intraocular inflammation including vitritis, retinitis or vasculitis. The diagnosis of CSD has been established on the basis of history of exposure to cats, clinical signs and symptoms in addition to positive serology tests for *B. henselae* (17). 

Serologic testing for *B. henselae* has shown as highly sensitive and specific for immunocompetent patients (18). Polymerase chain reaction has been recommended only for cases where the diagnosis remains suspicious or for immunocompromised patients (19). The diagnosis of ocular bartonellosis in our case was extremely likely, yet based on history of close contact with cats and a highly positive titer for *B. henselae* IgG. 

Cat-scratch disease is considered self-limiting, and treatment is recommended depending on the manifestation of the infection, the immune status and the patient’s age (20, 21). Our case did not receive any treatment due to the inactivity of the lesions and absence of any signs of intraocular inflammation. Besides, she was young and otherwise healthy. The case was followed by six monthly follow-ups for three years.

In summary, we describe unusual perivenous chorioretinal lesions associated with fibrous bands along the vessel wall; changes in vascular caliber and occlusion of the vessels make this case noteworthy what we recognized as a rare case of ocular bartonellosis with posterior segment manifestation in the absence of intraocular inflammation.

## CONCLUSIONS

*B. henselae* should be seriously considered in the differential diagnosis of perivascular chorioretinal lesions in the absence of intraocular inflammation.
